# Risk of Chronic Kidney Disease among Patients Developing Mild Renal Impairment during Tenofovir-Containing Antiretroviral Treatment

**DOI:** 10.1371/journal.pone.0162320

**Published:** 2016-09-15

**Authors:** Giuseppe Lapadula, Davide Paolo Bernasconi, Salvatore Casari, Franco Maggiolo, Roberto Cauda, Massimo Di Pietro, Nicoletta Ladisa, Laura Sighinolfi, Sarah Dal Zoppo, Francesca Sabbatini, Alessandro Soria, Chiara Pezzoli, Annalisa Mondi, Silvia Costarelli, Maria Grazia Valsecchi, Carlo Torti, Andrea Gori, for the Italian MASTER cohort

**Affiliations:** 1 “San Gerardo” Hospital, Monza, Italy; 2 University of Milano-Bicocca, Milan, Italy; 3 University of Brescia, Brescia, Italy; 4 Papa Giovanni XXIII Hospital, Bergamo, Italy; 5 Università Cattolica del Sacro Cuore, Rome, Italy; 6 Ospedale S. Maria Annunziata, Florence, Italy; 7 Ospedale Policlinico, Bari, Italy; 8 Ospedale Sant’Anna, Ferrara, Italy; 9 Istituti Ospitalieri, Cremona, Italy; 10 University “Magna Graecia”, Catanzaro, Italy; Temple University, UNITED STATES

## Abstract

**Background:**

Tenofovir (TDF) can cause kidney injury through tubular dysfunction, with or without drop of estimated glomerular filtration rate (eGFR). Whether mild eGFR reductions during treatment should be considered a reason for prompt TDF discontinuation, however, remains unclear.

**Methods:**

Patients with normal pre-TDF eGFR levels, who had developed mild renal impairment (i.e., two consecutive eGFR results between 89–60 ml/min) on TDF, were observed until onset of chronic kidney disease (CKD), defined as two eGFR<60 ml/min 3 to 6 months apart. Multivariable Poisson regression analysis was used to investigate whether outcome was associated with current and cumulative use of TDF (modeled as time-varying covariates).

**Results:**

2023 (29%) out of 6984 patients developed mild renal impairment on TDF. Among them, 191 progressed to CKD. The incidence of CKD did not significantly differ during TDF treatment (2.6 per 100 PYFU; 95%CI 2.2–3.2) or after its discontinuation (2.2 per 100 PYFU; 95%CI 1.8–2.6). However, the rate of CKD was significantly higher among patients continuing with TDF treatment compared to those who had discontinued it within 6 months of occurrence of mild renal impairment (aIRR 4, 95%CI 2.4–6.8). In contrast, among patients who had maintained TDF >6 months despite mild renal impairment, current TDF use was not associated with a significantly higher rate of CKD. Other significant predictors of CKD were older age, intravenous drug use, diabetes, hypertension, lower pre-TDF eGFR, higher eGFR drop since TDF introduction and longer exposure to TDF.

**Conclusions:**

Prompt discontinuation of TDF among patients developing mild renal impairment may prevent further progression of renal damage.

## Background

Tenofovir disoproxil fumarate (TDF) is widely used as a component of combination antiretroviral treatment (cART). The main adverse event associated with the use of TDF is nephrotoxicity with tubular dysfunction, with or without the reduction of glomerular filtration rate (GFR). Because TDF is excreted mainly in unchanged form by the kidneys, its clearance is significantly reduced in patients with moderate or severe renal impairment. In such patients, TDF use is preferentially avoided.[1] However, few studies have explored the safety of TDF in patients experiencing mild GFR reduction (i.e., between 89 and 60 ml/min). Whether, under these circumstances, TDF should be discontinued, remains a topic of debate. Previous observational studies have yielded conflicting results about the risk of progressive renal damage owing to prolonged exposure to TDF. Some studies have demonstrated an association between cumulative exposure to TDF and risk of chronic kidney disease (CKD), even among patients with normal renal function before TDF introduction.[2–4], Other studies, however, have suggested that loss in kidney function occurs mostly during the first year of TDF exposure and that risk of progression is relatively mild in the long term.[5,6] Adding to the uncertainty, a reduction in risk of kidney disease events after TDF discontinuation was found in some studies, but not in others. [3,4]

Hence, the best course to take in treating patients who develop mild renal impairment whilse on TDF has yet to be determined. As a consequence, the decision of whether to replace or suspend TDF often is up to the personal judgment of the physician.

Aims of our study were to evaluate the incidence of chronic kidney disease among patients who developed mild renal impairment during TDF treatment, to assess the risk of evolution to CKD while continuing TDF treatment compared versus discontinuing it and, lastly, to identify factors associated with the evolution to CKD.

## Methods

Patients were recruited from the observational Italian MASTER database cohort.

The MASTER cohort study was approved by the Ethics Committee of each participating center (Comitato Etico Provinciale della Provincia di Brescia, Comitato Etico della Provincia di Bergamo, Comitato Etico dell'Azienda Ospedaliera Policlinico Consorziale di Bari, Comitato Etico per la sperimentazione clinica dei medicinali dell'azienda sanitaria di Firenze, Comitato Etico della Provincia di Ferrara, Comitato Etico della Provincia Monza Brianza, Comitato Etico dell'Azienda Ospedaliera Istituti Ospitalieri di Cremona, Comitato Etico dell'Università del Sacro Cuore—Policlinico Universitario Agostino Gemelli di Roma). Written informed consent is obtained from the participants to the cohort upon enrollment. No specific consent was obtained for the present analysis.

Detailed description of the MASTER cohort is contained elsewhere.[7] Briefly, it is an ongoing prospective multicentre cohort which includes all patients in care for HIV infection in selected Italian clinical centers who have provided written informed consent to include their clinical and biological data in the MASTER database for scientific purposes. Patients are routinely seen every 3–4 months, and demographic, laboratory, clinical and treatment information are collected during each visit. Data are collected using a common electronic database, utilized by each participating centre for clinical purposes, and data is centralized, merged and checked for consistency on a 6-month basis. The database used for the present analysis was frozen in November 2014 and includes data from 8 clinical centers: Bari, Brescia, Bergamo, Cremona, Ferrara, Florence, Monza and Rome.HIV-1 infected patients who, between 2002 and 2013 had begun TDF treatment for the first time as part of an antiretroviral regimen were selected from the cohort if:

- estimated GFR (eGFR) before TDF introduction was >90 ml/min and
- they had developed mild renal impairment, defined as two consecutive eGFR between 89 and 60 ml/min, during TDF.

Estimated GFR was calculated using the Chronic Kidney Disease Epidemiology Collaboration (CKD-EPI) formula.[8]

Incidence rates of CKD, defined as two eGFR <60 ml/min measured 3 to 6 months apart, was calculated as events per 100 patient-years of follow-up (PYFU), together with relative 95% confidence intervals, based on an exact Poisson distribution. A Poisson regression model was fitted to estimate the association between current TDF use and incidence of CKD. Follow-up accrued from the second of the two consecutive eGFR defining mild renal impairment (study baseline) up to the development of CKD, or to the last available eGFR measurement. A multivariable analysis was carried out adjusting for several time-fixed factors: age, gender, country of birth, risk factor for HIV transmission, hepatitis C virus (HCV) co-infection (defined as serum reactivity for HCV-RNA or HCV-antibody positivity with unknown HCV-RNA), hepatitis B virus (HBV) surface antigen positivity, diagnosis of diabetes or hypertension, history of exposure to antiretroviral drugs prior to TDF introduction, use of boosted protease inhibitors, CD4+ T-cell count and eGFR preceding TDF introduction, eGFR drop between TDF introduction and occurrence of mild renal impairment, and cumulative exposure to TDF at the time of occurrence of mild renal impairment. Additionally, in order to investigate whether the effects of TDF current use might vary in relation to cumulative exposure to TDF since onset of mild renal impairment, we included an interaction term between these two time-dependent variables. The latter of these two variables was divided into four categories (<6 months, 6–12 months, 1–2 years, >2 years)

An additional analysis was carried out, in order to assess the impact of TDF discontinuation on yearly decline of eGFR. A multivariate linear model was fitted, using Generalized Estimating Equations method, to account for the clustered structure of data, i.e. time-dependent eGFR measurement within patients. This model was adjusted for the same time-fixed covariates listed above.

All statistical analyses were performed using R software version 3.2.2.

## Results

A total of 8,862 patients enrolled in the MASTER cohort initiated TDF for the first time, between January 1^st^ 2002 and December 31^st^ 2013. We excluded 1,575 patients whose eGFR before TDF was <90 ml/min, 229 who lacked eGFR determinations prior to TDF, and an additional 74 who lacked creatinine follow-up after TDF initiation. Among the 6,984 patients who had initiated cART regimens which included TDF when their eGFR was >90 ml/min, 2,023 (29%) developed mild renal impairment (i.e., two consecutive eGFR between 89 and 60 ml/min). These patients were therefore selected for all subsequent analyses. Most patients were male and Caucasians, with a mean age of 44 years (range 18–75). Their mean eGFR had dropped, after TDF introduction, from 104 to 80 ml/min. In about a quarter of the cases (494/2,023 = 24.4%), mild renal impairment was diagnosed within 6 months of TDF treatment. Table 1 illustrates, in detail, these occurences, as well as other characteristics at the time of the diagnosis of mild renal impairment. Subsequent to study inclusion, the median number of eGFR measurements per patient was 15 [inter-quartile range (IQR) 7–25], with a median gap of 3 months (IQR 2–4) between measurements. The median time between consecutive eGFR measurements did not differ significantly during TDF treatment and after its discontinuation (3 vs 2.9 months, P>0.05). MInimal correlation between average (within-patients) eGFR values and the frequency of eGFR measurements was observed (spearman rho = 0.24).

**Table 1:** Characteristics of patients developing mild renal impairment under TDF.

Characteristic	
**Gender,** male—N (%)	1396 (69.0)
**Age**, years—mean (sd)	44.1 (8.4)
**Place of birth**, N (%)	
Europe/North America	1930 (95.4)
Africa	50 (2.5)
Other	43 (2.1)
**HIV transmission route**, N (%)	
Heterosexual intercourse	781 (44.0)
Homosexual intercourse	186 (10.5)
Intravenous Drug use	618 (34.8)
Other/unreported	192 (10.8)
**History of AIDS defining events, N (%)**	522 (25.8%)
**Time since inclusion in the cohort, years–mean (sd)**	8.9 (7.2)
**HBsAg positivity**, N (%)	243 (12.0)
**HCVAb/HCV-RNA positivity**, N (%)	559 (27.6)
**Diabetes**, N (%)	194 (9.6)
**Hypertension**, N (%)	527 (26.1)
**Previously naïve to antiretroviral treatment**, N (%)	656 (32.4)
**Boosted-PI co-administration**, N (%)	1529 (75.6)
**CD4+ T-cell count, cells/μl–mean (sd)**	341 (239)
**Nadir CD4+ T-cell count, cells/μl–mean (sd)**	180 (136)
**HIV-RNA at TDF introduction, N (%**	
<50 copies/ml	686 (33.9)
51–1,000 copies/ml	196 (9.7)
1,001–10,000 copies/ml	206 (10.2)
10,001–100,000 copies/ml	474 (23.4)
>100,000 copies/ml	400 (19.8)
**Weight, kg–mean (sd)**	68.7 (13.6)
**eGFR before TDF introduction, ml/min–mean (sd)**	104.2 (9.2)
**eGFR drop since TDF introduction, ml/min–mean (sd)**	24 (13.7)
**Time on TDF, years**	3.6 (2.5)

eGFR was calculated using Chronic Kidney Disease Epidemiology Collaboration (CKD-EPI) formula.[8] Mild renal impairment was defined as two consecutive eGFR between 89 and 60 ml/min during TDF treatment. Abbreviations: eGFR, estimated glomerular filtration rate; HBsAg, Hepatitis B surface antigen; HCV-Ab, Hepatitis C virus antibodies; HCV-RNA, Hepatitis C ribonucleic acid; N, number; PI, protease inhibitor, sd, standard deviation, TDF, tenofovir disoproxil fumarate.

**Fig 1:**
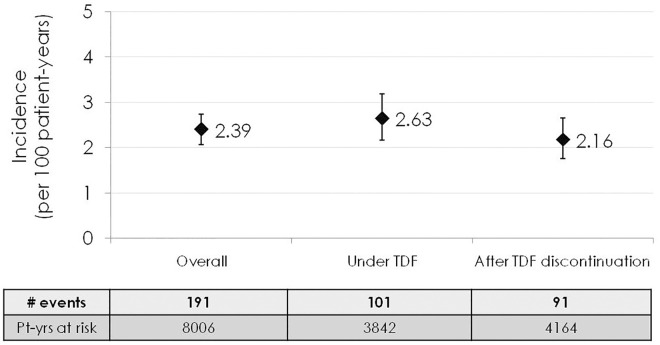
Incidence of chronic kidney disease among patients who developed mild renal impairment during tenofovir-containing antiretroviral treatment. eGFR was calculated using Chronic Kidney Disease Epidemiology Collaboration (CKD-EPI) formula.[8] Mild renal impairment was defined as two consecutive eGFR between 89 and 60 ml/min during TDF treatment. Chronic Kidney Disease was defined as two eGFR <60 ml/min measured 3–6 months apart. Abbreviations: Pt-yrs, patient-years; TDF, tenofovir disoproxil fumarate.

One hundred ninety-one out of 8,006 PYFU at risk subsequently developed CKD, accounting for an incidence of 2.39 events per 100 PYFU (95%CI 2.07–2.74). Of these, 101 developed CKD during TDF-containing cART, whilst 90, developed CKD after TDF, had already been discontinued, accounting for an incidence of 2.63 (95%CI 2.17–3.19) and 2.16 (95%CI 1.76–2.65) per 100 PYFU, respectively (Fig 1).

**Fig 2:**
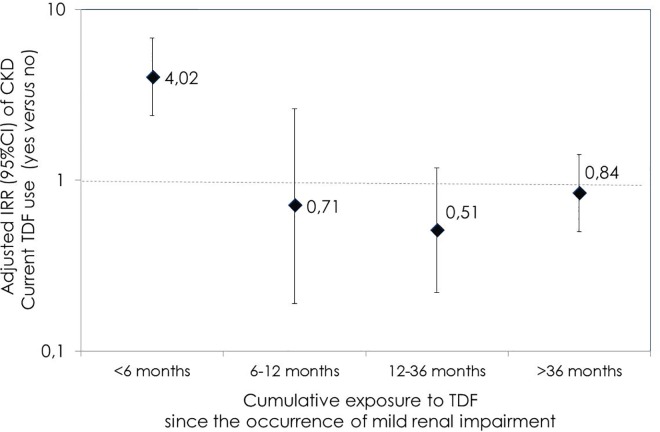
Association between current TDF use and CKD, based on cumulative exposure to TDF after mild renal impairment occurrence. eGFR was calculated using Chronic Kidney Disease Epidemiology Collaboration (CKD-EPI) formula.[8] Chronic Kidney Disease was defined as two eGFR <60 ml/min measured 3–6 months apart. Abbreviations: CI, confidence interval; CKD, chronic kidney disease; TDF, tenofovir disoproxil fumarate.

Overall, no statistically significant difference was observed when comparing the incidence rate of CKD during TDF with respect to the rate found after its discontinuation (incidence rate ratio [IRR]: 1.22; 95%CI 0.91–1.61). This result was confirmed using a multivariable analysis (adjusted IRR [aIRR] 1.12; 95%CI 0.79–1.59), adjusted for age, gender, country of birth, HIV risk factor, HCV and HBV coinfection, presence of diabetes or hypertension, baseline and pre-TDF eGFR, CD4+ cell count, previous ARV experience, use of boosted-PI and calendar year. Nonetheless, current use of TDF was associated with CKD when groups homogeneous for TDF exposure after onset of mild renal impairment were compared to each other. As shown in Fig 2, the rate of CKD was significantly higher among patients on TDF treatment regimens, compared to those who had discontinued it within 6 months since the diagnosis of mild renal impairment (aIRR 4.02, 95%CI 2.38–6.78). In contrast, current TDF use was not associated with a significantly higher risk of CKD among those patients who had stayed on TDF for more than 6 months after diagnosis of mild renal impairment, (aIRR 0.71 [95%CI 0.19–2.62], 0.51 [95%CI 0.22–1.18] and 0.84 [95%CI 0.5–1.42] among patients who had maintained TDF for 6–12 months, 1–2 years and >2 years after the diagnosis mild renal impairment, respectively).

Using multivariable analysis, the following factors were also significantly and independently associated with higher rate of CKD: older age (per 1 year increase, aIRR 1.06, 95%CI 1.03–1.09), intravenous drug use as risk factor for HIV acquisition (*versus* heterosexual risk factor, aIRR 2.27, 95%CI 1.38–3.73), diabetes (aIRR 1.58, 95%CI 1.02–2.44), hypertension (aIRR 1.61, 95%CI 1.11–2.31), higher eGFR drop between TDF introduction and study baseline (per ml/min lost, aIRR 1.07, 95%CI 1.06–1.09), and longer TDF exposure prior to onset of mild renal impairment (per year increase, aIRR 1.19, 95%CI 1.06–1.34). In contrast, higher eGFR before TDF introduction was associated with a significantly lower incidence of progression to CKD (per 1 ml/min increase, aIRR 0.94, 95%CI 0.92–0.97). No statistically significant effect was exerted by gender, country of origin, calendar year, hepatitis co-infection, co-administration of boosted-PI, previous ARV exposure and CD4+ count.

As illustrated in Table 2, the estimated annual rate of eGFR decline after TDF discontinuation was 2.6 (95%CI -0.91 to -4.3) ml/min/year. Patients stayng on TDF experienced an additional eGFR decline of 2.15 (95%CI -0.98 to -3.42) ml/min/year, corresponding to a mean eGFR reduction of 4.7 ml/min per year. Progression of renal impairment was seen to be at a lower rate in older patients with respect to their younger counterparts (eGFR per 1 year older: +0.15 [95%CI 0.07–0.23] ml/min/year), as well as in those patients who were taking TDF as part of their first cART, compared to those with previous exposure to antiretrovirals (+2.0 [95%CI 0.33–3.72] ml/min/year).

**Table 2:** Mean eGFR annual change (ml/min) among patients developing mild renal impairment during treatment with tenofovir. Results from the GEE multivariate linear model.

	Adjusted estimate	Lower 95% CI	Upper 95% CI	P
Intercept*	-2.6	-4.29	-0.91	0.003
**Male gender**	0.95	-0.51	2.4	0.202
**Age, per year**	0.15	0.07	0.23	<0.001
**Non-European heritage**	-0.29	-3.94	3.35	0.874
**Risk factor for HIV acquisition**				
**Heterosexual**	Ref.			
**Homosexual**	0.66	-1.77	3.10	0.593
**Intravenous drug abuse**	0.75	-1.05	2.55	0.416
**Other**	0.86	-0.74	2.46	0.292
**Current TDF use (*versus* discontinued)**	-2.16	-3.42	-0.90	<0.001
**Pre-TDF eGFR, per mL/min**	-0.06	-0.17	0.05	0.277
**Calendar year at TDF introduction**				
**Before 2005**	Ref.			
**2005–2006**	-1.71	-3.18	-0.24	0.022
**After 2006**	-1.95	-3.62	-0.28	0.022
**HBsAg positivity**	0.041	-1.84	1.92	0.966
**HCVAb/HCV-RNA positivity**	0.31	-1.39	2.01	0.72
**Diabetes**	0.58	-1.28	2.45	0.539
**Hypertension**	1.00	-0.42	2.43	0.167
**Naïve to ARV (vs experienced)**	2.02	0.33	3.72	0.019
**CD4+ before TDF introduction, per 10 cell/mm**^**3**^	0.02	-0.003	0.05	0.082
**Boosted-PI coadministration**	-0.05	-1.28	1.19	0.943

* Mean eGFR annual change (ml/min) for the reference patient (with average age, Pre-TDF eGFR and CD4+ before TDF introduction).

A multivariable Poisson regression model, aimed at assessing discontinuation rates of TDF, showed that higher eGFR (per ml/min increase, aIRR 0.99, 95% CI 0.984–0.996) and older age (per 1 year increase, aIRR 0.99, 95% CI 0.979–0.999) were significantly and independently associated with a lower risk of TDF discontinuation. No statistically significant association was found between discontinuation rate of TDF and the other explored covariates, including HIV risk factor, hepatitis co-infection, diabetes, hypertension and eGFR before TDF initiation (data not shown).

Among the 101 patients who had developed CKD whilst on TDF treatment, 62 subsequently discontinued TDF. After TDF discontinuation, eGFR improved to >60 ml/min in 37 cases, and remained at 30–60 ml/min or <30 ml/min in 20 and 5 cases, respectively. Follow-up after CKD development was available for only 52/90 patients who had developed CKD after TDF discontinuation. Among these, the last available eGFR was >60 ml/min, 30–60 ml/min and <30 ml/min in 19, 28 and 5 cases, respectively.

## Discussion

In this observational study of patients with previously normal eGFR, almost one third of the patients developed mild renal impairment during TDF-containing treatment. Further progression to CKD was, however, relatively rare, occurring in <3 cases per 100 patient-years of follow-up, despite the presence of established mild renal impairment. Previous randomized controlled trials of TDF reported very low prevalence of renal toxicity and, although small differences in glomerular filtration rate over time were noted, eGFR decline associated with TDF use was not considered to be clinically relevant.[9–11] Similarly, a systematic review and meta-analysis suggested that TDF-containing regimens were associated with a significantly greater loss of eGFR than regimens not containing TDF, although the magnitude of the effect was modest in clinical terms.[12] Nonetheless, several observational studies have demonstrated an association between TDF use and an increased risk of CKD, even among patients with normal renal function prior to cART initiation. [2–4,13] Whether initial mild kidney-function deterioration during TDF treatment are likely to worsen or stay relatively stable over time is unknown. Certain studies have suggested that prolonged TDF exposure increases risk of eGFR decline. [2,4,14] In other studies the loss in eGFR attributable to TDF was observed mainly during the first 6–12 months of exposure, and remained relatively constant afterwards.[5,15] The significance of subtle eGFR changes during TDF is, therefore, difficult to interpret in the clinical setting; hence the need to discontinue TDF when mild eGFR reduction is observed remains unknown.

In our cohort, switching away from TDF was associated with a lower risk of further progression of renal damage to CKD, but only if the switch occurred within 6 months from onset of mild renal impairment. In other words, among patients who were kept on TDF for more than 6 months despite the presence of mild renal impairment, a delayed TDF discontinuation did not apparently affect subsequent eGFR decline. Tenofovir toxicity is principally exerted on the proximal tubule, where it can accumulate and cause progressive mitochondrial DNA depletion and oxidative respiratory chain dysfunctions, ultimately leading to degenerative changes and cell apoptosis.[16,17] Among patients with mild renal impairment, initial modest eGFR reductions are likely to reflect, at least in part, impairment of active creatinine secretion across the tubule, rather than a reduction of glomerular filtration.[15] One possible explanation of our findings is that these tubule alterations are more likely to regress if the insult is withdrawn on a timely basis. However, if the discontinuation of the drug is delayed, progressive tubule loss and tissue scarring, and eventual damage to other renal structures (e.g. the glomeruli), can occur, turning a transient damage into a permanent impairment. Once irreversible damage is established, belated treatment switches may fail to significantly reduce the risk of CKD. Consistent with this hypothesis, previous reports have showed that a significant proportion of patients suffering from acute renal failure associated with TDF fail to acheive incomplete recovery of eGFR after TDF discontinuation.[18–20] Moreover, a more prolonged duration of TDF treatment prior to discontinuation has been associated with lower chances of renal function recovery.[18,20–22]. Of note, prolonged use of TDF at low eGFR is associated with higher levels of exposure to tenofovir, which leads to a more rapid progression of kidney function decline.[23,24] Early treatment switches to alternative drugs could prevent such a self-perpetrating mechanism of injury.

A second possible explanation for our findings is that drug-related reductions of eGFR progress more rapidly than those secondary to other common renal insults, such as hypertension or diabetes. Therefore, TDF discontinuation could be effective to prevent the damage directly related to the drug itself only if the treatment is changed in a timely manner. Conversely, a less rapid or relatively stable reduction of eGFR, despite prolonged TDF exposure, is more likely to be due to other causes and is hence less influenced by changes in the antiretroviral regimen. We cannot exclude, however, that higher rates of TDF discontinuation in patients with higher risk of CKD and/or a steeper eGFR decline, may have diluted the effects of TDF, thereby justifying the lack of association found between TDF use and CKD in patients exposed to TDF for >6 months. Previous large observational studies have failed to demonstrate that TDF exposure is an independent predictor of confirmed eGFR <60 ml/min among patients with normal pre-TDF eGFR, mainly because proactive switches away from TDF were observed when eGFR declined below 70 ml/min. [3] For similar reasons, no significant association between TDF use and end-stage renal disease was demonstrated.[25] In our cohort, an independent association between current eGFR and the rate of TDF discontinuation before CKD was observed. This could have led to underestimation of the risk associated with TDF use.

In any case, observance of mild renal impairment during treatment with TDF should prompt an intensive and rapid work-up, and, if other amendable causes of renal damage can be excluded, a rapid discontinuation of TDF should be effected. A timely TDF withdrawal may, in fact, reduce the risk of progression to CKD.

Our study does have some limitations that need to be acknowledged. First, our database lacked data on urine analysis, proteinuria, serum phosphate or other markers of tubular damage. Hence, renal damage evaluation was based solely on eGFR, which may not be the best marker of TDF-induced kidney toxicity. In addition, we were unable to adjust our analysis for co-administration of other nephrotoxic drugs, presence of acute comorbid conditions or family history of renal diseases. These factors may have acted as unmeasured confounders. Similarly, we could not control for genetic factors which may result in increased susceptibility to tenofovir-induced renal damage.[26,27] As previously mentioned, switches from TDF did not occur at random, but were clinician-driven; this may have led to underestimation of the risk of CKD among patients who were kept on TDF despite mild renal impairment, as these patients could have been perceived to be at lower risk of CKD. Finally, the majority of patients in our cohort were Caucasian; hence,our findings may not be directly transferable to cohorts with individuals of other heritage. Nonetheless, the large number of patients with previously normal eGFR who developed mild renal impairment under TDF is a strength of our study, as is the prolonged follow-up duration.

In conclusion, our study provides evidence that timely discontinuation of TDF (i.e., within 6 months of commencement) minimizes the risk of CKD among patients who have experienced mild eGFR reduction during treatment. Given the availability of alternative treatment options, a cautious approach in individuals with declining eGFR levels is therefore recommended.
